# Voices of the Costa Rican scientific diaspora: Policy lessons from a decade of experiences from our scientists abroad

**DOI:** 10.3389/frma.2022.904029

**Published:** 2022-12-08

**Authors:** Maria Esteli Jarquin-Solis, Enrique Lin-Shiao, Melania Guerra, Karlissa Calderón Zúñiga, Dayana Mora Solórzano, José María Gutiérrez

**Affiliations:** ^1^International Network for Government Science Advice, San José, Costa Rica; ^2^Cystic Fibrosis Foundation, Bethesda, MD, United States; ^3^Department of Environment and Development, University for Peace, Ciudad Colón, Costa Rica; ^4^Independent Researcher, San José, Costa Rica; ^5^Academia Nacional de Ciencias, Costa Rica, San Pedro, San José, Costa Rica; ^6^Instituto Clodomiro Picado, Facultad de Microbiología, Universidad de Costa Rica, San José, Costa Rica

**Keywords:** scientific diasporas, science diplomacy, Costa Rica, Latin America, research and development (R&D), higher education institutions, policy and investment strategies

## Abstract

Scientific diasporas have been identified as valuable resources to strengthen science, technology, and innovation in their countries of origin. In this context, our paper seeks to contribute by addressing the following research questions: What are the main features of the Costa Rican scientific diaspora, and what policy lessons can be extracted from their experiences abroad? Toward this goal, we analyzed ten years of diaspora perspectives as collected by TicoTal, an online database and network of Costa Rican scientists studying and working abroad created by the National Academy of Sciences (ANC) in 2010. Our study reveals the main features of the Costa Rican scientific diaspora using 121 interviews published over a ten-year period: we identified the academic areas in which the diaspora has specialized, the countries where they were trained, their current location, the most frequent funding mechanisms and sources that enabled professional opportunities abroad, the level of engagement and collaboration they maintain with the Costa Rican STI ecosystem, along with the incentives they consider important to support and harness the potential of this community to advance STI goals in the country. Results from this analysis can inform national policies and investment strategies in R&D infrastructure and resources, by providing a roadmap to engage with scientific diasporas and benefit from their training and talent, as well as guide future scholarship and exchange programs.

## Introduction

The migration of human capital from low-income countries to higher-income economies (known as “brain drain”) has been shown to affect the local consolidation of science, technology, and innovation (STI) in low and mid-income countries and can have significant impacts in specific fields (Meyer and Brown-Luthango, 1999; Commander et al., 2004; Giannoccolo, 2009; Kim et al., 2013; Vega-Muñoz et al., 2021). More recently, ‘scientific diasporas', defined as highly qualified human capital temporarily or permanently residing outside of their home countries, have been identified as a valuable resource to strengthen STI in their countries of origin (World Bank Institute, 2006). Indeed, the landscape of migration of talent has been described as one of ‘brain circulation', offering new possibilities to mobilize scientific diasporas as dynamic engines in support of the development of STI (Kuznetsov and Sabel, 2006; Tejada, 2007; Docquier and Rapoport, 2012; Siekierski et al., 2018; Shin and Moon, 2018; Organización Internacional para las Migraciones [OIM], 2019; Biglari et al., 2022). In this context, understanding the characteristics of scientific diasporas is imperative to strengthening national STI goals and programs.

Within this frame of reference, our paper seeks to contribute by addressing the following research questions: What are the main features of the Costa Rican scientific diaspora, and what policy lessons can be extracted from their experiences abroad? Toward this goal, we analyzed 10 years of diaspora perspectives as collected by TicoTal^1^, an online database and network of Costa Rican scientists studying and working abroad.

TicoTal^2^ (*Red de Talento Costarricense en el Extranjero*; Network of Costa Rican Talent Abroad) was created by the National Academy of Sciences (ANC) in 2010. This database compiles information from Costa Rican scientists and engineers who study or work abroad either permanently or temporarily. The main goal of TicoTal is to connect this diaspora with the Costa Rican STI establishment in order to foster alliances and cooperation that can strengthen and advance STI goals in the country.

Every month, ANC publishes an interview with a member of the TicoTal network, which is shared publicly *via* a listserv with all subscribers. Each interview covers the interviewee's pathway to STI opportunities abroad and highlights key challenges faced, incentives they value in the consideration to return or to collaborate with Costa Rican-based STI stakeholders, as well as perceived needs to bolster STI in the country.

A second platform that quantitatively tracks the Costa Rican scientific diaspora is *Hipatia*^3^, hosting a series of surveys, data analysis, and services covering the country's STI needs. Every year, *Hipatia* carries out a comprehensive and systematic mapping of the scientific diaspora. This resource contributes particularly by facilitating contact between Costa Ricans abroad and local startups so that they receive mentoring in different areas of expertise.

While these resources collect and maintain up-to-date information on Costa Rican scientists abroad, experts have emphasized the importance of analyzing the perspectives of scientific diasporas when designing policies of interest to these populations (Séguin et al., 2006; World Bank Institute, 2006). With this goal in mind, our paper consolidates the explicit perspectives of the Costa Rican scientific diaspora and carries out - for the first time—a mixed methods approach (systematic quantitative and qualitative analysis) of 10 years of interviews published by TicoTal between 2011 and 2021.

In the following sections, we analyze the academic areas in which the diaspora has specialized, the countries where they were trained, their current location, the most frequent funding mechanisms and sources that enabled professional opportunities abroad, the level of engagement and collaboration they maintain with the Costa Rican STI ecosystem, along with the incentives they consider important to support and harness the potential of this community to advance STI goals in Costa Rica. Lastly, we propose recommendations on how to generate engagement with an STI diaspora, in a way that positions members as key agents who favor the scientific and technological development of their home country.

## Methods

Our paper addresses the following research questions: What are the main features of the Costa Rican scientific diaspora and what policy lessons can be extracted from their experiences abroad? The main sources of analysis are the interviews “Talento Destacado” (hereafter called “Outstanding Talent”) published as part of the monthly ANC newsletter, with the purpose of highlighting Costa Rican students and researchers in STI fields who are living abroad. Their personal motivations and challenges, research interests, disposition, or preconditions to return home, and professional recommendations for Costa Rica to advance STI are valuable sources of information and encouragement for other young Costa Ricans who also wish to seek opportunities to study or work abroad. In addition, this platform also serves as a tool for members to connect among themselves across different parts of the world, learn about each other's research projects, and establish collaborative efforts.

The selection of the featured “Outstanding Talent” individuals is made by the ANC staff following no specified criteria, although gender equity is sought by alternating profiles from men and women. A sample of interviews was selected for this analysis, encompassing 121 interviews published in the newsletters between December 2011 and December 2021. The eight survey questions remained consistent across the decade covered by the analysis for all interviewees.^4^ Answers from interviewees were not word-limited and were not edited by ANC personnel prior to publication in the newsletter. This feature of survey consistency allowed for a systematic characterization of the diaspora.

The work of systematically reviewing these eight answers from 121 interviewees was divided among three of the coauthors (MEJS, ELS, and KCZ), who codified 62 variables out of these responses into a database. Variables were grouped into four main domains related to an interviewee: (a) identity and background, (b) funding conditions, (c) contact with Costa Rica, and (d) incentives to return and challenges abroad.

The first domain covers variables such as name, gender, month of the interview publication, and STI area of research, which can be chosen from a predetermined menu of options. Additionally, we extracted what undergraduate degree they reported and from which university it was obtained, as well as which country it is located in. We also codified if they reported having a Master's, a Ph.D., and/or a postdoctoral position (as binary variables), and the universities and countries each of these occurred in.

Identity and background variables were set on two temporal frames of reference: (i) the moment of the interview (recognizing there may be a 10-year time span between when this took place for different individuals) and (ii) the present, when the analysis is being performed (fixed as February 2022). Most identity and background variables are taken as reported at the moment of the interview. The few variables fixed in the present were extracted from other public sources, such as the interviewees' LinkedIn profile or their academic department's website. These updated variables include their current job position, the organization where they work, and the country of residence at present. Based on their full name, their birth date was extracted from the public database of the Costa Rican National Registry (Tribunal Supremo de Elecciones [TSE], 2022), in order to compute their exact age at the moment of the interview.

The second dimension of variables logs whether the individual's studies were financed through foreign, national (that is, Costa Rican) or personal funds, or a combination of these sources. Another variable related to funding codified if the financial sources were public or private money or a mix of the two. Finally, two variables registered whether the person reported having been part of an exchange or internship program abroad (binary variable), and the detail of how it served as a mechanism to enable the continuation of their studies.

Variables in the third grouping report if the person maintains contact with various sectors in Costa Rica, among them academia, industry, decision-makers, and colleagues in the diaspora (as binary variables). In the event that contact is reported, the detailed means of engagement is noted as an open, non-codified variable. This category also documents if the person is willing to establish a collaborative effort with Costa Rica (binary variable) and the details of that offering, for example hosting visiting students.

The final collection of variables registers the types of incentives the interviewees listed (as binary variables) as a precondition to returning to Costa Rica. They are grouped into three types:

- Infrastructure (relating to new research centers, equipment, access to information databases, salaries, or overall budgets).
- Networks (relating to training opportunities, scientific events, scientific collaborations, or positioning of science).
- Cultural (relating to the socio-economic and cultural conditions, other job opportunities or the role of science).

Open variables about the specific incentives requested from each category were annotated. Finally, there is a variable that registers, in a non-codified format, the challenges reported by the individual while abroad.

Given the relatively open frame of the interview questions, which in most cases do not specifically inquire about our target variables, we relied on the interviewees having directly reported each variable. The absence of a variable may simply reflect the failure of the person to document it in their answer, imposing a significant source of uncertainty on our analysis. Variables were not exclusively extracted out of predetermined questions, but in general variables about identity, background and funding were mentioned in the answers to questions 1. and 2., variables about contact with Costa Rica were predominantly inserted in answers 2. through 7., and variables about incentives were contained in answers 5. through 8.

It is also recognized that this study, by the original design of the raw dataset, is limited in scope to the Costa Rican scientific diaspora, which is defined as individuals who are studying and researching in areas from the natural and hard science fields. We acknowledge a significant gap in tracing and documenting valuable insights from the Costa Rican diaspora that work and study in the fields of social sciences, humanities, and arts. Furthermore, certain sample biases are implicit by way of the process through which the TicoTal network is generated. ANC relies in large part on mechanisms such as word of mouth, friends of friends, and media visibility to recruit new TicoTal members, biasing the pool of people from which the “Outstanding Talent” profile is then “hand-picked,” adding yet another bias. Some results presented here, for example, geographical representation may reflect these biases, as ties with certain groups of the diaspora are stronger due to geographical access or cultural proximity. Another limitation is that the data was collected over a 10-year period. It is therefore likely that interviewees may have been impacted by specific events and circumstances at the time of the interview, which may mean significant change between interviews conducted a decade ago compared to more recent ones. This has been addressed in the Results section with an analysis of responses over time.

The database was processed using Excel and R software, in order to compute statistics and trends and to generate the figures presented.

## Results and discussion

### A historical look at the consolidation of the STI community in Costa Rica

The development of STI in Costa Rica has been characterized by twists and turns since the dawn of its independent life in 1821. The first stages were primarily influenced by foreign naturalists, who studied the rich biodiversity of the country. Then, the appearance of local scientists seeded endogenous efforts in several fields, bolstered by liberal reforms that dominated the political landscape of the country in the last decades of the XIXth century and the beginnings of the XXth century, including the foundation of several research institutions focused on science and technology (Gómez and Savage, 1983; Coronado, 1997; Peraldo-Huertas, 2003; Solano Chaves and Díaz Bolaños, 2005).

A significant breakthrough in the institutional history of Costa Rica took place in 1940, with the creation of the University of Costa Rica (UCR). Besides offering novel opportunities for Costa Ricans to obtain professional degrees, scientific and technological research flourished in this institution in several fields over the next decades (Fallas-Santana et al., 2018). UCR, together with other more recently established public universities, has become the main reservoir of STI development in the country (Lomonte and Ainsworth, 2002; Sáenz-León and Rodríguez-Ramos, 2022).

Parallel key developments included the organization of a governmental framework for the promotion of STI in Costa Rica. The creation of the National Council for Scientific and Technological Research (CONICIT) in 1972 marked a turning point, as part of regional efforts in Latin America promoted by international organizations such as the United Nations Educational, Scientific and Cultural Organization (UNESCO), the Organization of American States (OAS) and the Inter-American Development Bank (IBD) (Sagasti, 2011).

Building on this progress, the Ministry of Science and Technology (currently Ministry of Science, Innovation, Technology and Telecommunications, MICITT) was created in 1986, the National Academy of Sciences (ANC) in 1992, and the Costa Rican Promoter of Innovation and Research in 2021, among other important developments (Fernández-Rojas, 2021). Additionally, the country has established national programs of STI, the last of which was recently issued for the period 2022–2027 (Ministerio de Ciencia, Innovación, Tecnología y Telecomunicaciones, 2021).

The establishment of a local STI community was also enriched by cadres of Costa Ricans who studied abroad in various disciplines and returned to work locally. In the 1950s UCR established an ambitious program of scholarships for graduate programs abroad which contributed to the consolidation of an academic community in this institution.

It is worth noting the example of Leonardo Mata Jiménez, a renowned microbiologist who obtained a Ph.D. at the Harvard School of Public Health and worked for over a decade at the Central America and Panama Institute of Nutrition (INCAP). In 1974, his return to Costa Rica was actively promoted by CONICIT and the UCR. As part of his return, a new research institute (Institute for Research in Health–INISA) was created at UCR in 1975 and Mata Jiménez was appointed as its first Director. He remained in the country for the rest of his career. Similar cases occurred in different areas of knowledge, thus resulting in the creation of different research centers and the strengthening of others.

Despite these developments, the country has significant limitations in incorporating STI as a dynamic element in its social and economic development. A detailed diagnosis of STI in Costa Rica was presented by the “State of the Nation program” in 2014 (Programa Estado de la Nación, 2014). This document highlighted important limitations in this area, such as: (a) low investment in research and development (below 0.4% of the GDP; Ministerio de Ciencia, Innovación, Tecnología y Telecomunicaciones, 2019); (b) limited generation of scientific and technological knowledge, as judged by the number of publications in journals and patents issued to Costa Ricans; (c) scarce links between the scientific and technological community and the broader local social and economic universe; and (d) a small community of researchers, which has problems of redundancy, gender gaps, generational relief, and academic inbreeding.

Given these circumstances, harnessing the potential of the Costa Rican scientific diaspora constitutes an additional opportunity to consolidate a robust STI community in the country (Séguin et al., 2006; World Bank Institute, 2006). The following sections will address the main features of this diaspora and identify policy lessons from their own perceptions and experiences abroad. We further contextualize these perspectives within the framework and recommendations proposed in previous literature on scientific diasporas and international research training.

### The Costa Rican scientific diaspora: A snapshot

This section aims to define who and where the Costa Rican scientific diaspora is (as captured by the TicoTal “Outstanding Talent” interviews), particularly what their academic trajectories have been and what funding mechanisms and network resources they used to train abroad. The analysis shows that 53% of interviewees identified as male, and the average age at the time of the interview was 36 years (age range: 23–64). At the moment of the interview, the vast majority (57%) of interviewees had already obtained a Ph.D., 34% were in the process of pursuing doctoral studies and an added 9% held a Master's degree but were not pursuing an additional degree. Notably, 11 individuals departed Costa Rica for STI employment opportunities abroad and did not pursue any further graduate studies.

**Figure 1:**
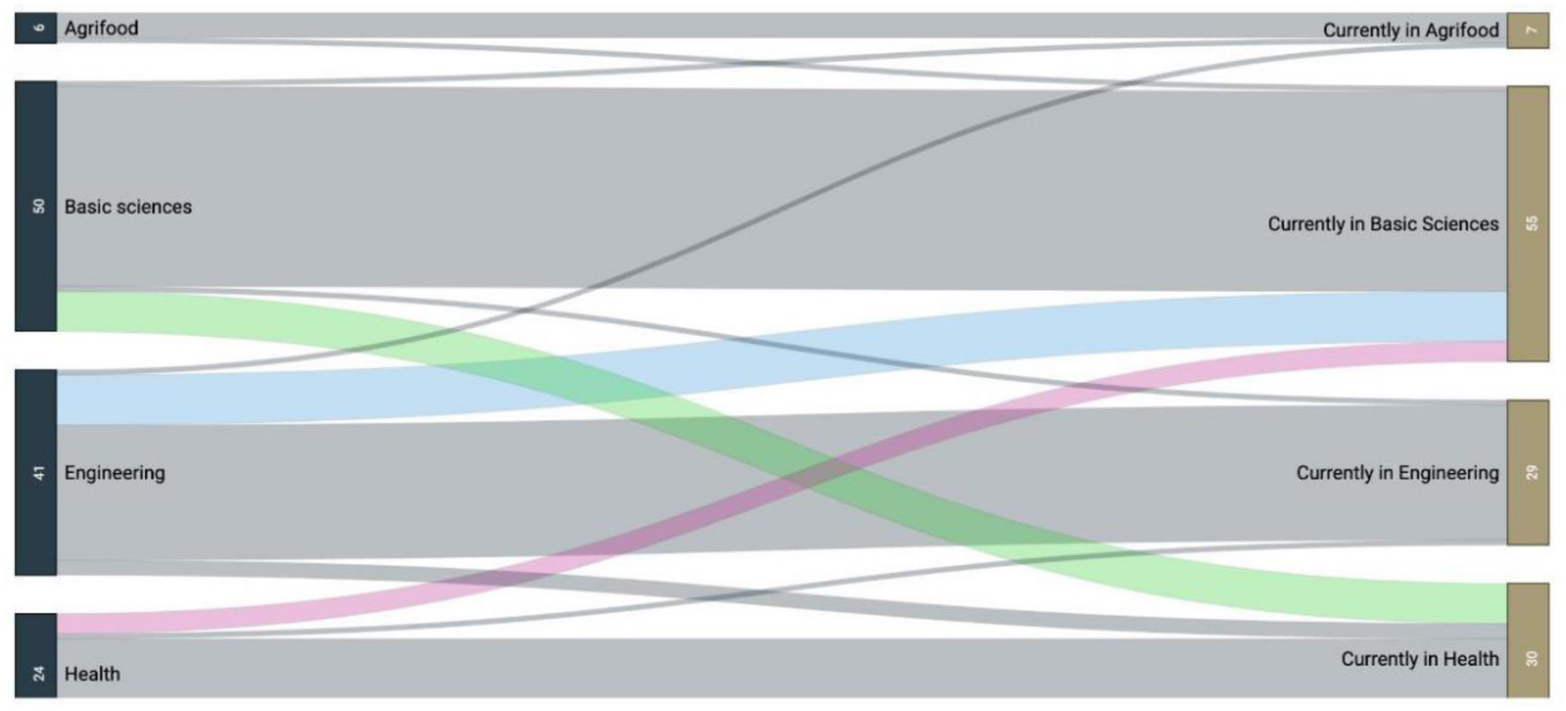
STI field of undergraduate studies (left column) and current STI fields in which members of the diaspora are working or training (right column). Numbers on the vertical boxes indicate individuals out of the total sample (*N* = 121).

In terms of STI fields, 41% of the sample were originally trained in basic sciences, 34% in engineering, 20% in health sciences and medicine, and 5% in food sciences at the undergraduate level (Figure 1). However, this image also shows that a subset of the diaspora changed their STI field when moving from their undergraduate level into their current field of work or graduate studies, especially migrating out of engineering to basic sciences, or from basic sciences to health. One hypothesis that might explain these transitions is that the diaspora finds increased flexibility and areas of interdisciplinary work when studying abroad. Further qualitative studies with larger samples should be conducted to confirm this.

**Figure 2:**
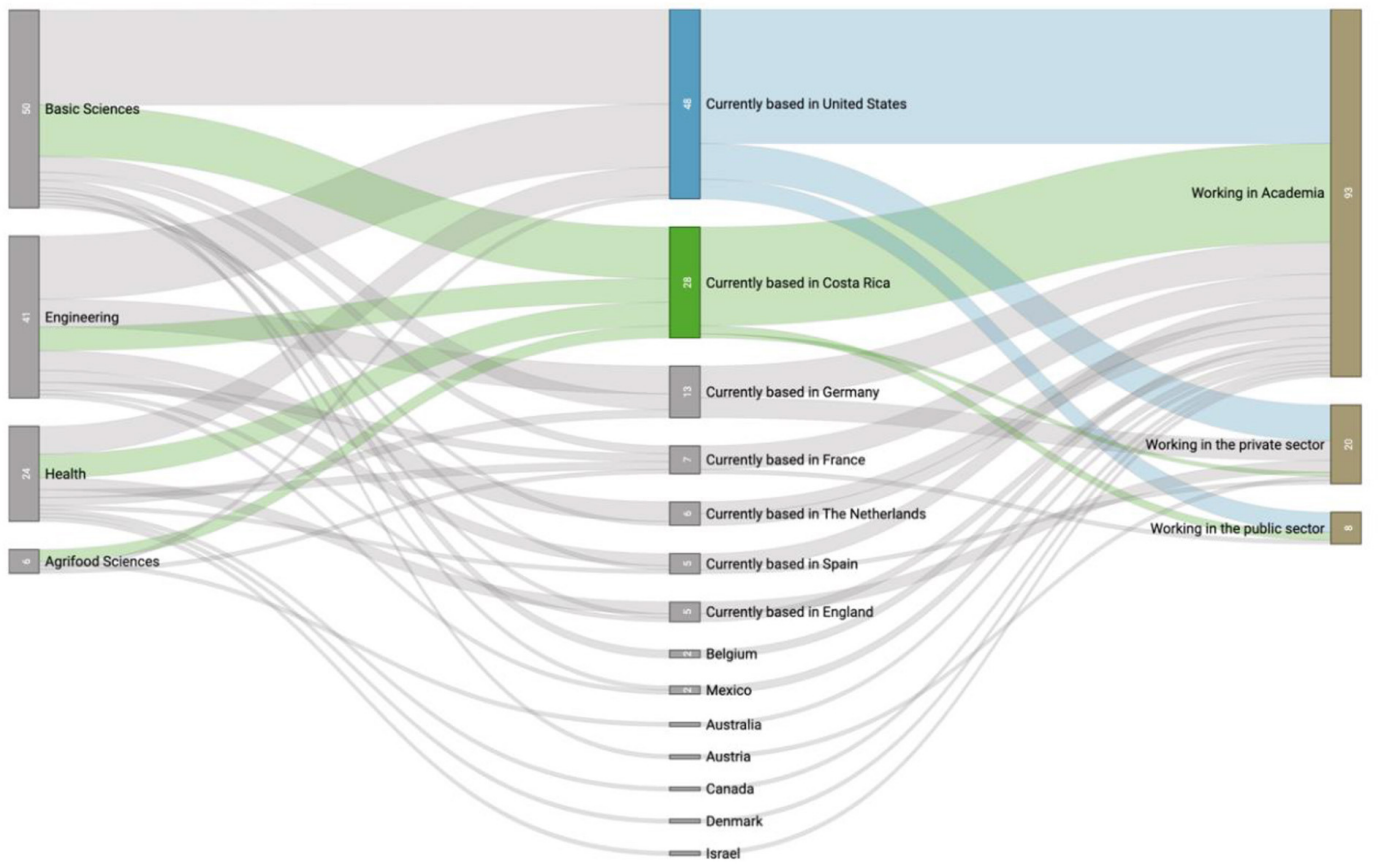
Current geographic location (middle column) and employment sector (right column) of the Costa Rican scientific diaspora by academic area (left column). Numbers on the vertical boxes indicate individuals out of the total sample (*N* = 121).

We also aimed to answer what proportion of the diaspora returned to Costa Rica over the 10 years covered by our sample. Our data shows that only 23% of interviewees returned by the time of analysis (2022). More specifically, Figure 2 shows that < 25% of the diaspora originally trained in basic and health sciences returned to the country. For engineering trainees, the percentage was even lower, with only 15% currently residing in Costa Rica (see flows in green). Together, our findings reveal low return and retention rates across all the STI areas covered by our data. A possible explanation is a lack of support via public policy and mechanisms aimed at attracting the diaspora back to the country.

In contrast, other Latin American governments have deployed official programs to repatriate their diasporas. Two well-known and well-characterized repatriation programs are RAICES (Network of Argentine Researchers and Scientists Abroad) and the CONACyT Repatriation Program in Mexico. Both have been in place for more than 20 years and focus on repatriating scientists, especially in the early stages of their careers, when they are doing their postdoctoral research (Rivero and Trejo-Peña, 2020). Thailand and Poland are also examples of countries that have implemented programs targeting highly qualified expatriates with the aim of encouraging them to transfer part of their scientific activity to their country of origin and promote technological development there (Meyer and Brown-Luthango, 1999; Szkarłat, 2020).

Another finding illustrated in Figure 2 is that half of the diaspora sample living abroad (48%) is currently based in the United States, and the vast majority were originally trained in basic sciences. A significant number of scientists are currently based in Germany, France, the Netherlands, Spain, and England (37%). These percentages are highly concordant with the geographical distribution identified by the *Hipatia* platform, which at the time of this analysis has published data on 759 members of the diaspora. In their dataset^5^, 70% of scientists report living in these countries, with the United States displaying the greatest presence (35%), followed by Germany (12%), Spain (8%), The Netherlands (6%), and United Kingdom (5%). France occupies the 7th position (4%), just behind Canada (5%), which is the only country with a limited presence in our sample. Other studies revealed that the Mexican scientific diaspora is also concentrated in these same countries and in Brazil (Marmolejo-Leyva et al., 2015).

Figure 2 also depicts the sector in which this sample is currently training or working. Of the 121 interviewees, 77% are working or continuing their training in academia, and only 23% have transitioned to jobs in the private and public sectors. Notably, while 56% of the interviewees residing abroad work in academia, a small number currently work in foreign public entities such as the National Aeronautics and Space Administration (NASA), the United States Patent and Trademark Office, and the Virgin Islands Department of Health. Additionally, private sector companies in the United States, Germany, and England have hired 21% of the Costa Rican diaspora, which is expected since industry has a high capacity to absorb the abilities that diaspora members offer, according to International Organization for Migration and Migration Policy Institute (2012).

These examples stand in contrast to interviewees who have returned to Costa Rica, as the vast majority work in academia (89%), a single person was identified working in the private sector, and two additional people in the public sector (a microbiologist in the national health system and a chemist as Director of Research and Development in the Ministry of Science and Technology at the time of the analysis). Further studies are required to analyze the current efforts by the country's public and private sectors to attract scientific talent from abroad. An example of a national plan that attracted researchers back to civil service and industry was an Argentinian plan, which brought back 178 scientists between 1992 and 1994. Among this group, the largest share joined the private sector (31%), while 15% opted for public administration jobs (Meyer, 2015).

**Figure 3:**
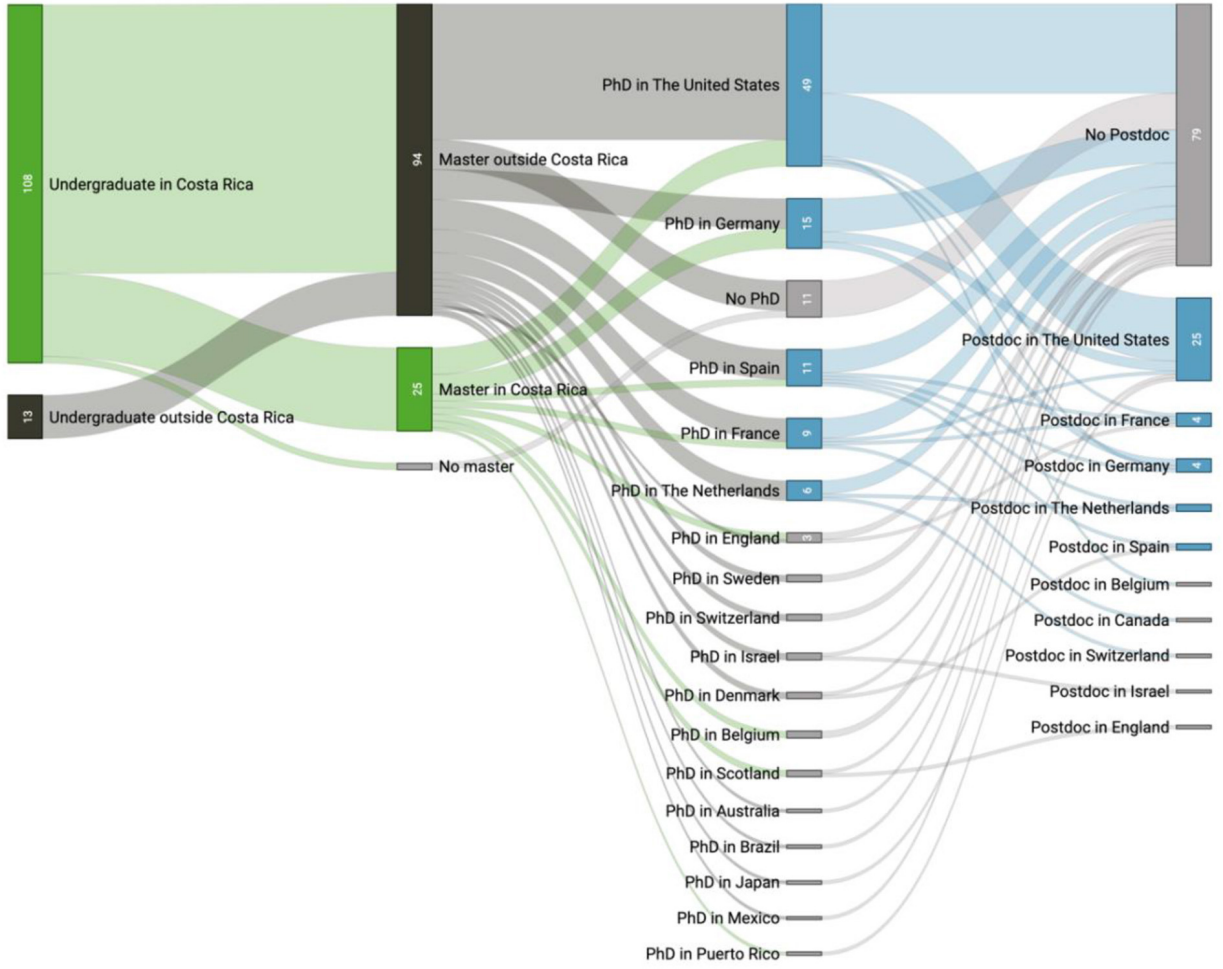
Academic pathways of the Costa Rican scientific diaspora: from undergraduate (left column) to postdoc (right column). Numbers on the vertical boxes indicate individuals out of the total sample (*N* = 121).

Another goal of our study was to determine in which countries the interviewees pursued their academic degrees and at what point they decided to go abroad. Figure 3 visualizes four academic time points of these 121 scientists: the country where they completed their undergraduate, Master's, and doctoral degrees or conducted postdoctoral training.

Figure 3 shows that the majority of interviewees (89%) obtained their undergraduate degrees in Costa Rica. Of these, only a small number continued their Master's studies within the country followed by a doctorate abroad (see flows in green). Two findings are striking here: (1) no one in this sample pursued their doctoral studies in Costa Rica, and (2) none of those who completed their undergraduate degree abroad returned to the country for graduate degrees. This phenomenon is explained by Dodani and LaPorte (2005), stating that expatriated citizens acquire highly specialized skills which are not frequently taught in their home countries. There is therefore an important challenge for universities: how to retain or attract internationally trained Costa Ricans back to their institutions, either for a graduate degree (Master's or Ph.D.) or to conduct postdoctoral work.

An additional finding of Figure 3 is that regardless of the area of expertise, the majority of people who completed a Ph.D. (82%) and a postdoc (88%) did their training in one of the following five countries: United States (US), Germany, Spain, France, and the Netherlands (see flows in blue). Postdocs are mainly concentrated in the US and Europe, with a single person in Israel. This selection of a subset of countries could be an important guide for decision-makers in academia and government regarding where to deploy bilateral agreements to promote the mobility of Costa Rican scientists abroad; it also highlights where new agreements and collaborations can be improved or increased, to facilitate opportunities in other places.

A noteworthy finding of Figure 3 is that only 37% of the analyzed sample performed postdoctoral work. Significant efforts have been made in the country in recent years to finance postdoctoral fellows, and thus higher numbers of Costa Ricans pursuing postdoctoral training abroad are expected over the next years. Indeed, the UCR financed postdoctoral training abroad for 32 trainees from their institution in the last 5 years (Oficina de Asuntos Internacionales y Cooperación Externa [OAICE], 2022), and a new postdoctoral program was established at UCR just recently.

Another interesting aim of this study was to identify the funding sources used by Costa Ricans to carry out their training abroad. Of the 121 interviewees, 82% indicated that they mainly received financial support from public institutions, both foreign and national, with a large proportion directly funded by stipends provided by foreign public universities (61%)^6^.

Of note, 11 people were co-sponsored by public universities of the home and the host country. This could present an important framework to be included in future scientific and international cooperation agreements: The practice of co-financing the training of scientists abroad may in fact be attractive to host universities. Previous studies have shown that -contrary to the so-called “return brain drain”-, once these students return to their countries of origin, they become potential scientific linkages to the host institutions (Jonkers and Tijssen, 2008). In addition, the analysis further revealed that private financing is mainly provided by private universities overseas or direct family support and personal savings. Overall, this highlights little engagement by the private sector in providing and financing academic training opportunities for Costa Ricans abroad.

Lastly, our study sought to identify whether interviewees had used a specific mechanism as a springboard to facilitate their insertion into postgraduate programs abroad. Results show that 23% of people in our sample explicitly indicated having used one. Among the different mechanisms, the most commonly used were research internships (61%), exchange programs (21%), and established or past scientific collaborations between investigators in the home and the destination university (18%). This result is relevant for decision-makers in academia, as it highlights that financing short-term research internships constitutes a platform for students and researchers to obtain additional training opportunities and funding opportunities abroad. For example, the Turkish American Scholars and Scientists Association (TASSA) allocates resources to send students to higher education institutions in the United States to work with diaspora members in specific laboratories (Burns, 2013).

### Building bridges: Engagement with the home country and future incentives

**Figure 4:**
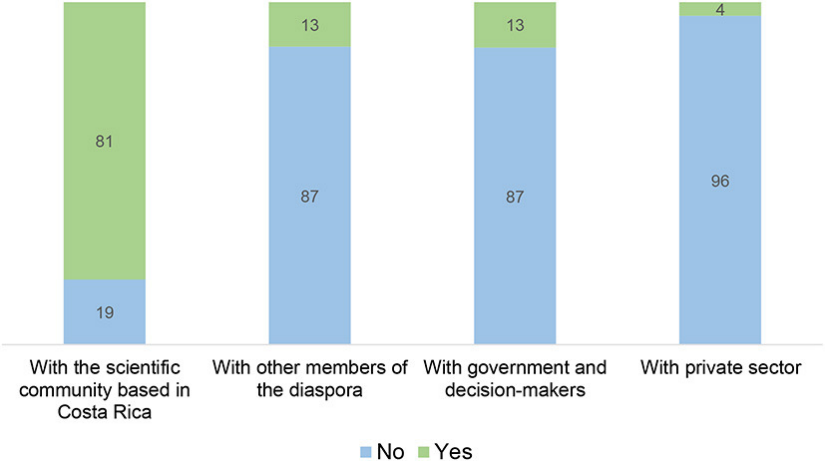
Percentage of members of the Costa Rican scientific diaspora who indicated collaborating with key stakeholders in the home country, by sector (*N* = 121).

This study performed a qualitative analysis regarding the type of social interactions that the interviewed members of the diaspora indicated having with the home country, and specifically with STI stakeholders. This is particularly relevant given that the specialized literature on scientific diasporas has highlighted the importance of creating academic networks between scientists abroad and their country of origin, as well as facilitating platforms where the diaspora can advise decision-makers and advance scientific agreements between the home country and the local ecosystem where they are based (Meyer and Brown-Luthango, 1999; World Bank Institute, 2006; Burns, 2013; Wren, 2018; Ittelson and Mauduit, 2019; Jarquín-Solís, 2022). Among the possible social interactions, our qualitative analysis identified the four STI stakeholders most commonly mentioned in the responses: the local scientific community, government and decision-makers, the private sector, and other members of the diaspora. Figure 4 displays the percentage of members of the diaspora who indicated collaborating with these actors.

As can be seen in Figure 4, the vast majority of the diaspora has had interactions with the broader Costa Rican scientific community (81%). In some cases, they were in permanent communication with former mentors or colleagues from their universities back home, underscoring an interest in staying current with the developments of these groups. In other instances, a closer relationship was established, including regular visits to Costa Rica to participate in seminars or workshops and active teaching in graduate programs in national universities. A few interviewees who did their Ph.D. studies abroad informed that a part of their research was carried out in their home country.

Some interviewees have promoted the development of internship programs for Costa Rican students in the universities where they study or work, and others have promoted the development of cooperation agreements and memorandums of understanding (MOUs) between foreign universities and academic institutions in the home country. This is the case of a scientist based at the Institut Pasteur in France, who has actively promoted cooperation and exchanges with the research community in the field of Microbiology, including a cooperation agreement between the UCR and this French institution.

Figure 4 also shows that only 13% of interviewees have had some interaction with other members of the diaspora, for example, one professor served as “Chair” of the doctoral thesis defense committee of a Costa Rican student at Stanford University, another person led a research project in the United States and invited other Costa Ricans to participate, and a group of people mentioned speaking as panelists at events organized by the diaspora abroad.

On a similar vein, only 13% of interviewees indicated having had some type of collaboration with the government or with decision-makers. A notable case is a scientist based in Europe who provided expert advice to the Costa Rican diplomatic delegation at the United Nations Framework Convention on Climate Change (UNFCCC). This example highlights the potential role of diaspora members in supporting diplomatic efforts, an especially critical function, considering that most Costa Rican embassies do not have a designated science and technology counselor (EURAXESS, 2022).

Diplomatic corps from Latin America are engaging with their diasporas in thriving ecosystems, as in the case of Boston, where the Consulate General of Mexico recruited 25 experts from the Mexican diaspora to form an advisory group that updates diplomatic personnel on the latest developments in STI. Something similar is being done by the Consulate General of Brazil, with the aim of connecting with its diaspora to promote the internationalization of Brazilian startups and companies in the United States (Ittelson and Mauduit, 2019). This illustrates the link between science and diplomacy in response to global challenges and countries' specific needs (Gluckman et al., 2017).

Our study provides other examples of interactions with the government, establishing collaborations with the national health system (the Costa Rican Social Security Fund or “CCSS”), the Central Bank, the National Statistics and Census Institute, and the ministries of agriculture and science and technology, as well as individuals who have served as delegate members in national elections organized by embassies and consulates abroad. While our analysis suggests limited connections between the Costa Rican government and its diaspora, this is not generalizable to other countries in Latin America. Mexico, for example, created the Institute for Mexicans Abroad and established a Consultative Council where elected diaspora members can make recommendations to the government on a wide range of issues (International Organization for Migration and Migration Policy Institute, 2012).

Finally, the interaction of the interviewed diaspora with the private sector in Costa Rica has been especially low (4%) and no concrete examples of collaboration with companies in the country were identified within our sample.

Despite the limited interaction with the private sector and other STI stakeholders, a key finding of our analysis is that there is a significant number of members of the diaspora who expressed an interest in maintaining contact and contributing scientifically to Costa Rica. Previous studies have highlighted that research on the needs and perceptions of the diasporas has been lacking and might be key to determining future policies directed at engaging with them (Séguin et al., 2006; Gëdeshi and King, 2019). To contribute to this specific gap, we analyzed the responses to four questions seeking recommendations from the diaspora on initiatives to support talent abroad, as well as incentives to retain scientific talent in the country and resolve the development needs of specific scientific areas. Our analysis uncovered three main incentives: (1) expanding research networks, (2) strengthening infrastructure, and (3) broadening cultural perception around STI.

Most of the interviewees highlighted that expanding research networks (92%) is a key incentive, including funding and expanding training opportunities abroad, promoting collaborations among the Costa Rican scientific community, organizing events, and promoting international relations and collaborations with higher education institutions abroad. These ideas were persistently recurrent throughout the 10-year period of interviews.

Some examples of specific demands in this area are: (1) the strengthening of existing or the creation of new databases with information about scientific talent abroad that can facilitate contact with ministries, as well as with public or private institutions and research groups; (2) the organization of online courses targeted to specific populations such as high school students, and (3) the establishment of pathways for the diaspora to receive Costa Rican students for internships and training.

Building scientific networks involving Costa Rican research groups and members of the diaspora is a key task that should be fostered by multiple mechanisms. These networks should be fluid and versatile, away from bureaucratic formalisms, and based on the shared interests of members of the Costa Rican scientific community, the diaspora, and groups from other countries in complex networking dynamics, along the spirit of what has been called “the new invisible college” (Wagner, 2008). The strengthening of academic “critical masses” in diverse STI fields constitutes an element of attraction to members of the diaspora, whether to return and work in the country or to establish novel cooperation bonds. Sustained efforts and policies should be promoted to consolidate such an international networking arena.

A significant number of interviewees mentioned incentives regarding the improvement and strengthening of infrastructure (86%). In particular, the majority of the diaspora demanded a higher budget allocation to STI, funded by both the government and the private sector. Strikingly, the budget-related demands remained constant regardless of the year in which the interview was conducted.^7^ Interviewees envision an ecosystem where academia, industry, and other sectors are more dynamically linked to foster attraction and further development of companies that carry out research and development (R&D), ultimately opening a broader set of opportunities that can attract members of the diaspora back to the country.

Further infrastructure incentives stated by interviewees -especially in the early years from 2011 to 2016- are the creation of research centers with modern equipment and access to databases that can keep up with development in the discipline, as well as programs and pathways for those scientists who wish to start a small business. It is interesting to note that requests for new physical infrastructure diminished considerably in more recent interviews. This may imply that there is a perception among the diaspora that the country has invested in this area in recent years.

Finally, there were additional demands related to the perception of science in society, as well as more job opportunities for the STI community and the redesign of the academic pathway within the country. In particular, 77% of people stated the importance of maintaining an updated listing of job offers in STI in Costa Rica, that is accessible to the diaspora. This need appeared continuously throughout the 10-year period. Additionally, many interviewees mentioned that clearer performance criteria and a merit-based academic system should be created.

The diaspora also considered that reinsertion initiatives for professionals abroad should be managed in a structured manner and as part of a directive national plan with a long-term vision, facilitating certain processes such as creating salary ranks that are based on both experience and academic degrees. The variety of needs expressed by the interviewees can be viewed within a more general frame of cultural and institutional changes aimed at raising the status of STI in the imaginary of our society as a whole, well beyond the STI community. This is a long-term goal that should be based on concerted national policies at various levels and involving many stakeholders.

Overall, these pressing demands call for a stronger commitment of public and private stakeholders to build a sustainable landscape for STI development, that not only will attract members of the diaspora, but will also retain young scientific talent by creating conditions for an expanding ground of international cooperation.

## Conclusions and policy recommendations

This study was developed with two key objectives. First, to understand the main features of the Costa Rican scientific diaspora using TicoTal's 121 interviews published over a 10-year period. The second goal was to extract policy lessons from the diaspora experiences abroad. We studied their perspectives, resulting in an analysis that can inform national policies and investment strategies in R&D infrastructure and resources. The following policy recommendations emerged from this analysis:

It is crucial to position scientific diasporas as key resources in the scientific and technological development of countries. They must be reflected as critical actors on a nation's short, medium, and long-term STI policies. Government institutions must perform a systematic and recurrent mapping of their diasporas and their capacities and, on that basis, promote active networking with the home community in STI.^8^

The latter has been China's strategy, i.e., positioning the diaspora as a national asset in its global policies. In the last 20 years, they increased opportunities for the diaspora by fostering collaboration, short stays, and joint appointments between Chinese scientists who work abroad and their local STI community. This agrees with the perspectives of the Costa Rican diaspora collected in these interviews. In fact, China's approach has been crucial to its global rise in multiple scientific fields (Vogel, 2011; Marginson, 2022). New technological advancements have further bolstered this process. Indeed, different studies have highlighted that China, India, Mexico, and South Korea benefited from using information and communication technologies with their US-based diaspora. This provided a considerable comparative advantage over nations that do not make use of these strategies (Patterson, 2005; Grossman, 2010).

In addition, as mentioned by interviewees, academia should foster knowledge circulation by developing a set of incentives to attract the diaspora back, either temporarily or permanently. For example, local universities could invite diaspora members to conduct workshops and short-term stays, as a way to build long-term relationships that can be converted into more permanent positions. Furthermore, as exhibited in the Results section, another challenge for local universities is to enroll diaspora members in graduate and postdoctoral programs offered within the country. Bolstering these programs could attract talent during the training process and be attractive to diaspora members, further strengthening brain circulation and mobility of ideas.

Expert literature has additionally emphasized the importance of hiring researchers from abroad as a key indicator for the internationalization of research institutions (European Science Foundation, 2012). On some fronts, Costa Rica may find it challenging to recruit researchers with no previous ties to the country, but these appointments would not necessarily have to be permanent; they could be transitory in order to promote an “open door model” that benefits circulation. In fact, a university in Brazil offers an institutional affiliation, through which a work contract is not generated but grants scientists institutional support (Bonilla, 2022).

Moreover, a set of elements should be taken into account when signing future international agreements between universities: First, incentivizing more co-sponsorships of graduate studies abroad between home and host universities as a way to strengthen scientific linkages between countries (Jonkers and Tijssen, 2008). Also, our study, as well as previous ones, have shown the importance of financing short-term research internships in key universities that could eventually be converted into additional funding or lower barriers to further graduate studies and research opportunities for scientists (Burns, 2013).

However, engagement with the diaspora should not be viewed as exclusive to academia. As highlighted by the interviews, there is a need to involve both public and private sectors which, in turn, will also benefit from the knowledge, networks, and national ecosystems where the diasporas are based. A report by International Organization for Migration and Migration Policy Institute (2012) underscored the importance of creating better bridges with the private sector, sharing resources, promoting increased R&D private investment, and engaging STI diasporas in international projects.

Public-private partnerships can be established to guide diaspora investments in their home country. Such is the case of the Philippines, where Business Advisory Circles have been founded comprised of industry, government, and non-government organizations. They advise Filipinos abroad on where and how to invest in their country of origin (International Organization for Migration and Migration Policy Institute, 2012). In a similar vein, public institutions related to STI should engage with the diaspora in order to strengthen their own research agenda and harness opportunities in the institutions where members of the diaspora study or work abroad.

This study has included several successful examples of diverse and innovative ways in which other countries have engaged with their scientific diasporas. Similar mechanisms to bolster the diaspora in Costa Rica could have the following results in the midterm: (1) Increased access to new technological developments and (2) Increased production and improved knowledge circulation across the local scientific community and industry in the country. In the long term, these strategies could result in the strengthening of the nation's bilateral and multilateral relations using science and technology as a vehicle, an objective at the very core of science diplomacy.

Our paper provides new evidence about scientific diasporas. The biggest contribution is that it fills a gap in the literature providing more research into the needs and unique perceptions of diasporas. Despite the limitations, this study provides valuable information in order to build a roadmap to engage with scientific diasporas and benefit from their training and talent. Future research is crucially needed to better grasp the current efforts deployed in both public and private sectors and assess their efficacy and impact. This represents many methodological challenges; however, efforts should be made to collect this information in order to expand on the evidence presented in this paper.

## Data availability statement

The raw data supporting the conclusions of this article will be made available by the authors, without undue reservation.

## Author contributions

MJ-S, MG, and EL-S designed the research and database. KC, MJ-S, and EL-S collected the data and prepared the database. MS made the figures. MS and JG drafted the first manuscript. MJ-S, JG, EL-S, MG, and DM contributed to the subsequent writing and editing of the manuscript. All authors contributed to the article and approved the submitted version.

## Footnotes

^1^It can be accessed *via* the following link: http://ticotal.cr/conozca-acerca-de-ticotal.html.

^2^At present, the TicoTal network has 556 members (who will be referred to here as “TicoTales”): 380 men and 176 women, who are distributed across 31 countries. Of them, 282 TicoTales reside in the American continent, 247 in Europe, 14 in Oceania, 12 in Asia and one in Africa. They are all active postgraduate students or professionals in STI fields, such as engineering, technology, agricultural, natural, and medical sciences.

^3^It can be accessed *via* the following link: https://hipatia.cr/.

^4^The interview included the following questions: (i) What were the determining factors in and/or opportunities to go abroad?, (ii) In general terms, how would you describe your living conditions abroad?, (iii) What type and intensity of professional contact do you have with CR?, (iv) Recommendations of initiatives that support talent abroad to act as agents of development in Science and Technology, (v) Would you be willing to return to the country if the right possibilities arise?, (vi) What incentives do you consider pertinent to retain scientific talent in the country?, (vii) In your area of work, what would be the development needs of the area in CR? and (viii) Would you like to make any additional comments?

^5^This data was downloaded in October 2022 and can be found here: https://hipatia.cr/dashboard/diaspora-cientifica.

^6^During fieldwork, it was only possible to locate the funding source for 93 people, which represents 77% of the analyzed sample.

^7^As stated in the methods sections, the data was collected over a ten-year period, which may mean significant changes between interviews conducted a decade ago compared to the most recent ones. For this reason, a comparison was made between the answers of the early and late interviews to understand whether some demands of the diaspora had evolved over time or whether they remained constant.

^8^The European Union Global Diaspora Facility is an initiative that is currently mapping diasporas around the world, in order to assist countries and diaspora organizations to collaborate more effectively with each other on development issues (Diaspora for Development, 2021). It can be accessed *via* the following link: https://diasporafordevelopment.eu/.
